# Review of Component Materials and Diverse Applications of Polymer Concrete

**DOI:** 10.3390/ma18122745

**Published:** 2025-06-11

**Authors:** Xiaolei Li, Jinyuan Gu, Yunsheng Xu, Shen Li, Rui Zhang

**Affiliations:** School of Civil Engineering and Architecture, Xi’an University of Technology, Xi’an 710048, China; civillxl@xaut.edu.cn (X.L.); 2230720058@stu.xaut.edu.cn (J.G.); 2230721154@stu.xaut.edu.cn (Y.X.); ruizh@xaut.edu.cn (R.Z.)

**Keywords:** polymer concrete, resin, aggregates, fillers, fibers, nanofillers, applications, properties

## Abstract

Polymer concrete (PC) refers to the use of a polymer as a replacement for cement, enhancing the mechanical and durability properties of traditional concrete. Introduced in the late 1950s and gaining prominence in the 1970s, the use of PCs has been rapidly increasing across various industries. This paper provides a comprehensive review, beginning with a brief historical overview of polymer concrete. It examines key review papers and books related to PC, summarizing the various materials commonly used in its formulation, such as resins, fillers, fibers, and nanofillers. Additionally, the paper explores the diverse applications of polymer concrete, ranging from structural repairs and architectural cladding to advanced uses in electrical insulation and 3D printing, with special attention given to sustainability aspects. Through this review, the paper highlights the growing importance of polymer concrete in modern construction and infrastructure projects.

## 1. Introduction

Polymer concrete (PC) has gained significant traction in the construction industry due to its superior properties compared to traditional concrete, such as higher compressive strength (ranging from 70 to 120 Mpa) and flexural strength; fast curing (achieves around 70−75% of its strength after a curing of only one day at room temperature); impervious to liquids; small number of pores; high freeze–thaw resistance resulting from its non-moisture-absorbing property; good electric insulation; high resistance to corrosive chemical substances, including acids and bases; high resistance to scratches; excellent vibration damping properties; et al. [1,2].

**Figure 1:**
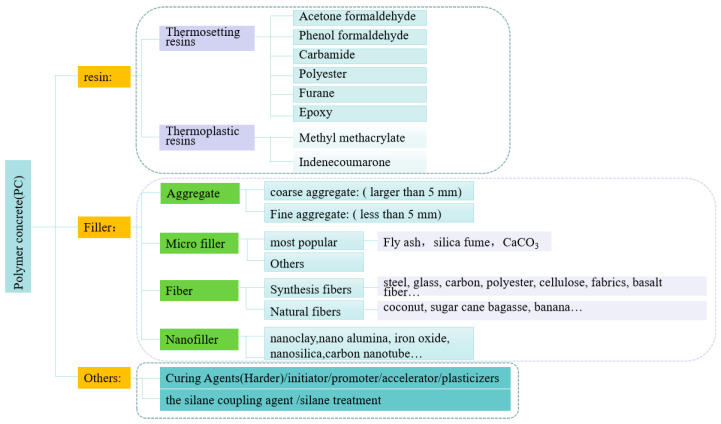
The common components of PC.

Based on principles of process technology, polymer concrete composites typically can be classified into three main categories: polymer concrete (PC), polymer-impregnated concrete (PIC), and polymer cement concrete (PCC) [3]. In some studies, a fourth category is included, distinguishing between normal or ordinary polymer concrete (OPC), polymer-modified concrete (PMC), polymer-impregnated concrete (PIC), and surface-coated polymer concrete [4]. Here, OPC is synonymous with PC, and PCC with PMC. From a materials science perspective, polymer concrete (PC) is a composite material primarily composed of synthesized polymers and aggregate; the aggregate is bound together in a matrix with a polymer binder, excluding Portland cement and water. The PC mixture also includes microfillers to fill the microvoids (spaces between the fine and coarse aggregates) and various fiber reinforcements to optimize the performance of polymer concrete products for specific applications. Nanoparticles serve as nanofillers in PC, providing extensive contact areas and preventing the formation of numerous subcritical microcavities and microcracks. Additionally, curing agents, initiators, and accelerators are incorporated into the mixture. According to [3], polymer resins are divided into two types: thermoset and thermoplastic. The common components of polymer concrete are depicted in Figure 1.

**Figure 2:**
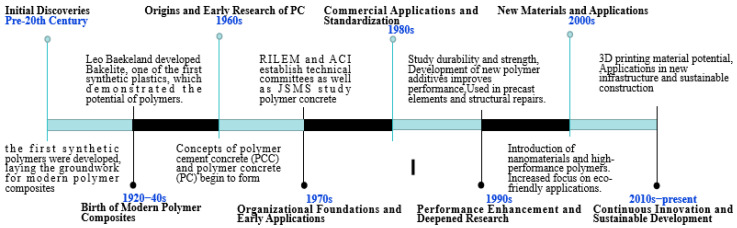
The developmental timeline of polymer concrete.

The technological development of polymer concrete originated in the early 20th century with the invention of synthetic polymers. Leo Baekeland’s development of Bakelite showed the potential of synthetic plastics, laying the groundwork for subsequent innovations. By the 1960s, concepts of polymer cement concrete (PCC) and polymer concrete (PC) began to take shape, marking the origins of this innovative material. During the 1970s, organizations such as RILEM and ACI established technical committees to study and standardize polymer concrete, facilitating its early applications, and then PC was used to repair and protect concrete structures. Standards and guidelines were developed, and studies focused on improving durability and strength through new polymer additives. After that, performance enhancements and deeper research were complemented, and nanomaterials and high-performance polymers were introduced with an increased focus on eco-friendly applications. Currently, a continuous push for innovation and sustainable development in the field of polymer concrete has been ongoing. The development of polymer concrete is shown in Figure 2.

From a technological development perspective, early research primarily focused on optimizing resin systems and improving mechanical properties [1,5,6,7,8,9]. Then, the incorporation of nanofillers, fiber reinforcements, and functional additives further expanded the multifunctionality and high-performance capabilities of PC [10,11,12,13,14,15]. Additionally, the growing emphasis on sustainability has prompted researchers to explore bio-based resins and recycled aggregates in polymer concrete to minimize the environmental impact [16,17,18,19,20].

Especially this year, since epoxy polymer concrete may encounter complex stress states in practical applications, triaxial compression tests were conducted to investigate the influence of confinement ratios on the mechanical properties and failure criteria of EPC [21]. The health monitoring of polymer concrete under varying temperatures was conducted using sinusoidal ultrasonic signals combined with an integrated artificial intelligence approach [22]. A mesoscopic random aggregate model was adopted to study the damage evolution and size effect of epoxy polymer concrete. The relationship between the size effect rate and the fractal dimension rate for epoxy polymer concrete samples was revealed [23]. The mechanical and microstructural performance of polymer permeable concrete made with polyester and epoxy resins and different aggregate types (calcite and basalt) under long-term acid exposure was investigated [24]. Polymer concrete, such as polymethyl methacrylate polymer concrete (PMMA-PC), can exhibit significantly higher tensile strength [25]. The bending response of a polyester resin reinforced by marble powder, silica, and sand grains was analyzed. In addition, a model using artificial neural networks (ANNs) was also proposed to predict the combinations of each constituent that lead to maximizing the mechanical properties of PCs [26].

Despite extensive research, challenges remain in material design, process optimization, and the long-term performance evaluation of polymer concrete. Key issues include interfacial compatibility between resin matrices and aggregates, volumetric stability during curing, and degradation behavior under high temperatures or UV exposure. Furthermore, advancements in 3D printing and smart materials present new opportunities for PC in complex structural fabrication and functional integration [27,28,29], yet they also impose stricter requirements on rheological properties and processability.

This paper provides a systematic review of the research progress and application status of polymer concrete. Beginning with a historical overview, it conducts a detailed analysis of existing scholarly research on its constituent materials, including key components such as resin, aggregate, micro filler, fibers, nanofiller, and various agents. Building upon this foundation, the paper thoroughly examines the material’s practical applications in traditional engineering fields such as structural repairs, architectural cladding, and electrical insulation, as well as its innovative uses in emerging technologies like 3D printing. Notably, this study analyzes the sustainable development of polymer concrete from two critical perspectives: reducing landfill dependency and lowering carbon emissions. Finally, based on current research status and technological needs, constructive prospects for future research directions of polymer concrete are proposed. Through this comprehensive review, the paper aims to provide references for material innovation and sustainable development research in polymer concrete while promoting its wider application in modern construction engineering.

## 2. Commonly Used Components/Materials of Polymer Concrete

**Figure 3:**
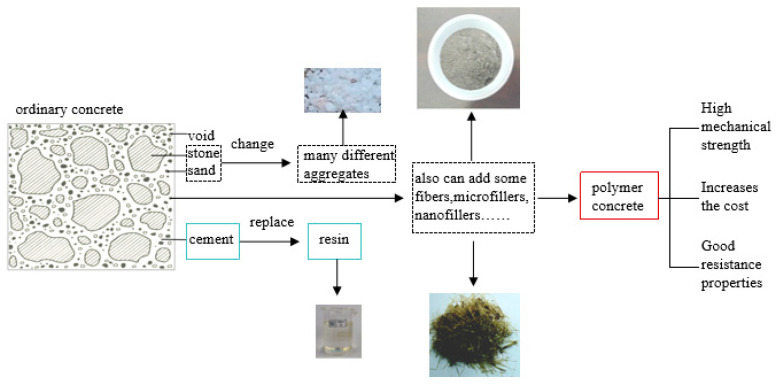
Transition from ordinary concrete to polymer concrete.

In polymer concrete (PC), the conventional cement binder is substituted with resins, as illustrated in Figure 3. This substitution offers numerous advantages to the material. By replacing cement with resin, PC exhibits enhanced properties, such as high strength and durability, accelerated curing times, excellent chemical stability in harsh environments, superior resistance to water ingress, good resistance to freeze–thaw cycles, and low electrical conductivity. Furthermore, besides resins, the aggregate components, like stone and sand, can also be modified by adding various fibers and nanomaterials to further reinforce PC.

### 2.1. Resin

In addition to the aforementioned advantages, the use of resins in polymer concrete (PC) also introduces some disadvantages. Thermoset resins are highly cross-linked polymers, whereas thermoplastic resins remain uncross-linked. Thermoplastic polymers soften and flow when heated, which makes them unsuitable for structural applications. Therefore, thermoset resins are predominantly utilized in PCs. Table 1 lists the most commonly used polymeric resins, along with their respective drawbacks.

Each resin has its own characteristics, and when used to make polymer concrete, its own properties and the correlations with other aggregate or fillers vary greatly. To modify resin properties, such as reducing the high shrinkage characteristic of vinyl ester resins, methyl methacrylate (MMA) is commonly incorporated into the binder system at concentrations of 2.5~5 wt.%. It is also noteworthy that for unsaturated polyester, in addition to commercial unsaturated polyester, many researchers have achieved the chemical transformation of recycled PET into unsaturated polyester, which has been applied as a PC binder.

### 2.2. Filler

#### 2.2.1. Aggregate

**Figure 4:**
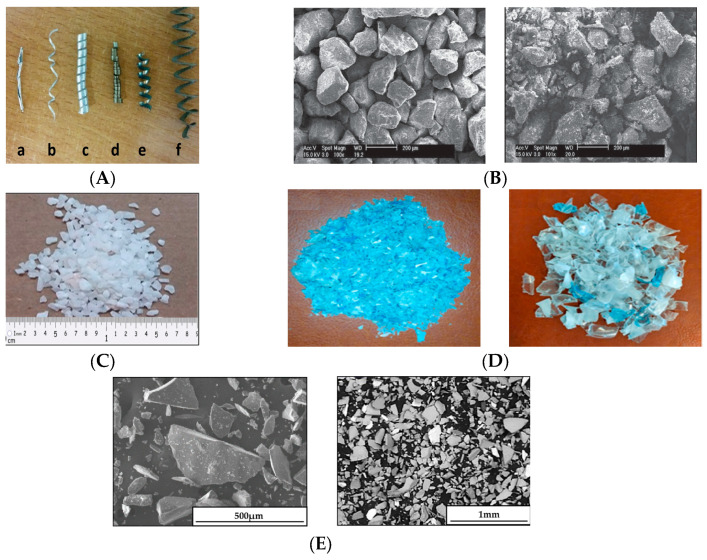
Some of the common aggregates of PC. (**A**) a–f shows titanium; aluminum fine chip; aluminum medium chip; and steel fine, medium, and thick chip [17]. (**B**) SEM micrographs of crushed basalt aggregate and silica sand aggregates [37]. (**C**) Marble particles [36]. (**D**) Fine recycled PET and coarse recycled PET [16]. (**E**) Micrographs of coarse RGA and fine RGA [38].

Various types of aggregate materials have been used by researchers, with most choices based on locally available materials to reduce costs. The most commonly used aggregates include river sand, Ottawa sand, silica sand, blasting sand, quartz, crushed stone, crushed granite, marble [36], and crushed basalt [37]. Some commonly used aggregates in PC are illustrated in Figure 4.

In recent years, the release of industrial waste into the environment due to advancing technology has caused significant damage to both the environment and human health. Consequently, many industrial wastes have been used to replace natural aggregates in polymer concrete. Examples include industrial waste machining chips [17], plastic waste, waste tires, recycled PET bottles [16], wasted construction materials, GFRP waste, waste Tetra Pak particles, metallurgical wastes, industrial waste (palm oil fuel ash and red mud) [39], recycled glass aggregate (RGA) deriving from cathode ray tube (CRT) glass waste [38], and so on. These materials not only help in reducing environmental impact but also contribute to the cost-effectiveness and sustainability of polymer concrete.

#### 2.2.2. Microfillers

Microfillers are introduced into the polymer concrete (PC) mixture to fill the spaces between the fine and coarse aggregates. These microfillers are fine powders with particle sizes of less than 80 microns. Incorporating microfillers into PC reduces the total void volume and average pore size, thereby enhancing the physical and mechanical properties of the material. Additionally, using microfillers decreases the overall fabrication cost of PC by reducing resin consumption.

The most commonly used microfillers include calcium carbonate, fly ash, quartz powder, and silica fume. Utilizing waste and natural materials as microfillers is advantageous for the circular economy. Some effective green microfillers for polymer concrete are agricultural waste of palm oil fuel ash (POFA) [40], waste Ground Glass Fiber [41], rapid-cooled steel slag (RCSS) obtainable from industrial byproducts [42], biochar—which should not be used as a fuel [19], quartz powder [32], Singkut leaf plants [43], and thermoset composite wastes.

**Figure 5:**
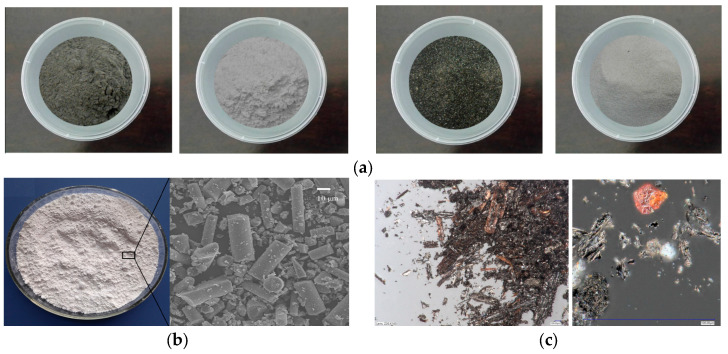
Some of the common microfillers of PC. (**a**) Ground palm oil fuel ash (GPOFA), calcium carbonate, unground palm oil fuel ash (UPOFA), and silica sand [40]. (**b**) Photograph of the GGF particles and SEM image of the GGF particles [41]. (**c**) Optical microscope view of overall picture of biochar and shape of a single grain [19].

These materials not only improve the sustainability of polymer concrete but also make use of waste products that would otherwise contribute to environmental pollution. Some commonly used microfillers in PC are illustrated in Figure 5.

#### 2.2.3. Fiber

**Figure 6:**
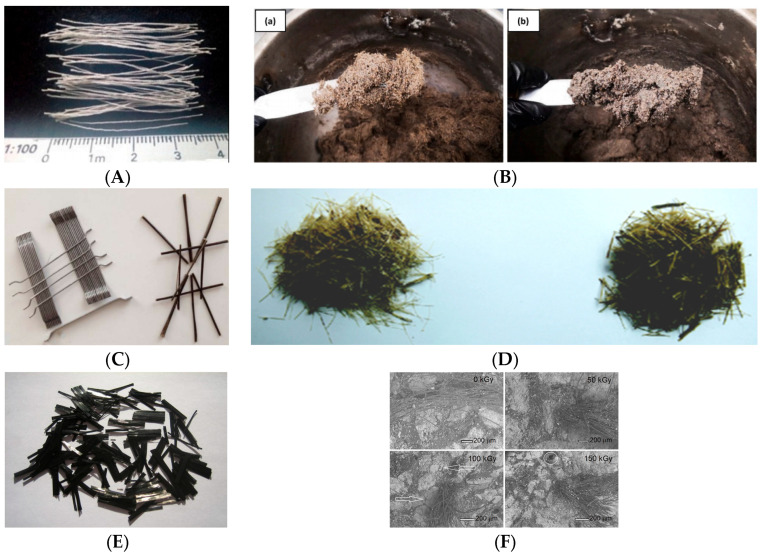
The common fibers of PC. (**A**) Polyester fibers [36]. (**B**) PC with polyvinyl alcohol fiber and carbon fiber (a) mixed 15%-10/90-PVA, and (b) mixed 15%-10/90-Crb [18]. (**C**) Steel and basalt fibers [48]. (**D**) Chopped sisal fibers and ramie fibers [49]. (**E**) Short-cut carbon fibers [45]. (**F**) SEM images of PC with polyester fibers [50].

A significant body of research has investigated the reinforcement of polymer concrete (PC) through the addition of various types of fibers. The inclusion of fibers can significantly enhance the strength and toughness of polymer concrete. Fibers used in PC can be broadly classified into two types: natural and synthetic fibers. Some commonly used fibers in PC are illustrated in Figure 6.

Synthetic fibers commonly used in polymer concrete include steel fibers, basalt fibers [44], glass fibers, carbon fibers [45], polyvinyl alcohol (PVA) fibers [18,46], nylon fibers, polyester fibers, cellulose, fabrics, polypropylene fibers [47], polyurethane resin fibers, 3D printed fibers [27,28]. These fibers have been added to polymer concrete in varying quantities to improve its properties. For example, PVA fibers can enhance the tensile strength and toughness of PC, while basalt fibers contribute to high-temperature resistance.

Natural fibers used in polymer concrete include coconut, sugar cane bagasse, banana [51], kenaf, palm, flax, wool, hemp [52], sisal fibers, ramie fibers [49], corn husk fibers [53], vegetable fibers [54], coffee shell fibers [55], and so on. Furthermore, some natural fires with resistant lateral flakes, such as Stipa Tenacissima and Stipa Pennata, are able to bring additional microstructural reticulation of the concrete, increasing the mechanical properties [56]. Natural fibers offer several advantages, such as renewability, low cost, abundance, non-abrasive characteristics, and lower health and safety concerns compared to synthetic fibers. However, while the addition of fibers generally improves the overall performance of polymer concrete, studies have shown that it can enhance some properties while potentially reducing others.

#### 2.2.4. Nanofiller

**Figure 7:**
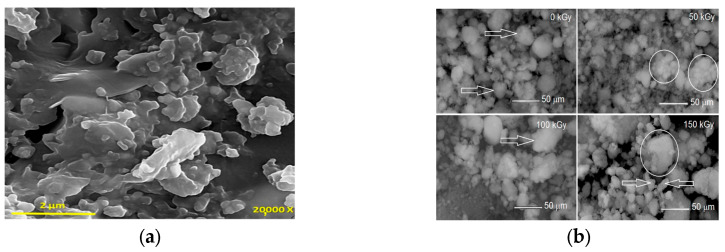
The common nanofillers of PC. (**a**) SEM micrograph of BFPC-clay2 sample [13]. (**b**) SEM images of nanosilica after irradiation [57].

In recent years, researchers have explored the use of nanomaterials to enhance the properties of concrete and polymer composites. Nanomaterials offer significantly higher surface areas than microfillers and additives, allowing them to interact at multiple scales and alter performance at the mesoscale. As nanofillers, nanoparticles provide extensive contact areas and help prevent the formation of numerous subcritical microcavities and microcracks. While the use of nanoparticles in concrete on a large scale may not be economical, even small amounts of nanomaterials can significantly improve the mechanical properties of polymer concrete (PC), including strength, ductility, and fracture toughness. Various nanomaterials have been incorporated into PCs, such as nano clay, nano alumina, carbon nanotubes, iron oxide, and nanosilica. Some commonly used nanofillers in PC are illustrated in Figure 7.

Nano clay: The most widely used nanofiller in PC research is nano clay. It is worth noting that ceramic waste slurries have a lot of nano clay particles, which might be very hazardous if they are not properly neutralized and recycled; this was evidenced by the presence of nano kaolinite by atomic force microscopy (AFM) in ceramic tile wastewater slurry [58]. Nano clay enhances compressive, flexural, and impact strength, as well as thermal stability. However, it may reduce the tensile strength of PC [59]. Nano clay particles cause crack deviation and create rough fracture surfaces, requiring more energy for crack propagation and thereby increasing fracture energy [13]. Alumina nanoparticles (ANPs): These significantly improve the ductility and fracture toughness of epoxy PC compared to neat PC [12]. PCs using montmorillonite-unsaturated polyester (MMT-UP) nanocomposites show higher compressive strength, elastic modulus, and splitting tensile strength than those using pure UP [60]. Carbon nanotubes (CNTs): These reduce curing time and enhance mechanical properties compared to reference PCs. Functionalized CNTs yield higher compressive strength than unfunctionalized ones [15]. PCs with 1.0 wt% multi-walled carbon nanotubes (MWCNTs) introduced by the weight of epoxy resin show a 36% improvement in energy absorption compared to conventional PCs [61]. Silica nanoparticles (SNPs): Adding nanosilica to PC can improve bonding strength [62]. However, at low nanosilica content and high radiation doses, the material exhibits high deformation, producing ductile PC but with lower compressive strength than control specimens [57]. Hybrid mixtures: Using a combination of pristine and carboxyl (COOH) functionalized MWCNTs can produce a very ductile PC with appreciable tensile strength. Experimental investigations reveal that COOH functionalization maximizes PC ductility, achieving failure strains of up to 5.5% and increasing toughness by 184% [14].

In the study referenced [63], molecular dynamics (MD) simulations were used to analyze the shearing behavior of carbon nanoparticle (CNP)-reinforced PC composites. MD simulations allow for atomic-level insights into the interactions between CNPs and the polymer matrix, providing a more detailed understanding of how surface-modified CNPs enhance mechanical properties. In reference [64], nanoparticles, including Multi-Walled Carbon Nanotubes (MWCNTs), Aluminum Nanoparticles (ANPs), and Silica Nanoparticles (SNPs), were added to an epoxy-based PC to examine how the nanoparticles affect the bond strength of PC compared to a steel substrate. The result shows that Aluminum Nanoparticles (ANPs) provided the best improvement in bond strength. Multi-Walled Carbon Nanotubes (MWCNTs) showed very limited to no increase in bond strength. Silica Nanoparticles (SNPs) resulted in a general decrease in bond strength compared to neat PCs. These enhancements in polymer concrete through nanofillers contribute significantly to the advancement of high-performance construction materials.

However, it should be noted that in addition to the high cost of nanomaterials, there is a major problem with using nanoscale fillers in polymer concrete, which is the difficulty of dispersing them in the binder. Due to their high surface energy, nanoparticles tend to agglomerate, especially at concentrations exceeding 1 wt.%, leading to inhomogeneous material properties. Additionally, the large difference in thermal expansion coefficients between the polymer matrix and nanofillers can induce internal stresses, potentially compromising long-term structural integrity. Without proper treatment, nanoparticle agglomeration can create weak interfacial zones, reducing composite performance.

To address these dispersion challenges, researchers have explored various methods to improve nanoparticle dispersion through combining primary physical dispersion methods with auxiliary surface modification techniques, including high-energy ultrasonication (both probe and bath variants), mechanical mixing (shear mixing and magnetic stirring), and hybrid strategies that integrate multiple techniques (such as sequential magnetic stirring and ultrasonication), complemented by surface functionalization methods like silane coupling agents and plasma treatment [50], which collectively enhance nanoparticle dispersion through synergistic physical and chemical interactions.

### 2.3. Others

Besides resin and fillers, other additives, such as curing agents, hardeners, initiators, catalysts, accelerators, and plasticizers, are also included in polymer concrete (PC) to achieve specific properties. Silane treatment or the addition of silane coupling agents is primarily used to enhance interfacial bonding performance, improve water and chemical resistance, and enhance mechanical properties [65].

#### 2.3.1. Curing Agents (Harder)/Initiator/Promoter/Accelerator/Plasticizers

An accelerator is a chemical used to increase the rate of cure in a free radical system by reacting with the initiator. A catalyst is a substance that markedly speeds up the curing of a binder when added in minor quantities. An initiator is a substance capable of causing the polymerization of a monomer by a chain reaction mechanism, often incorrectly called a catalyst. Promoters are reducing agent compounds added to the monomer system to cause the decomposition of the peroxide initiators in the system, and they are often referred to as accelerators. Some agents used in polymer concrete are shown in Table 2.

The initiator can adjust the viscosity of the original resin, not only affecting mechanical and durability properties but also adjusting the setting time, strength growth rate, thermal expansion coefficient, shrinkage, and compatibility with other surrounding materials. Common families of curing agents and initiators used in the literature include ones from the aliphatic and aromatic amines, anhydrides, polyamides, polysulfides, and mercaptans, as well as the catalytic and latent hardeners family [4].

Methyl ethyl ketone peroxide (MEKP) is a widely used initiator. Studies show that the amount of MEKP significantly affects the mechanical properties of PCs but does not influence failure modes and load transfer patterns [76]. Cross-linking agents trimethylolpropane trimethacrylate and tetraethylene glycol diacrylate are usually used for acrylic resins. Oligomeric silsesquioxanes, hydroxyethyl methacrylate polyurethanes, and others are recommended to be considered as additives to the binder in polymer concretes. Moreover, 3-methacryloxypropyltrimethoxysilane is used as a coupling agent for polyester [6].

Plasticizers are used to improve the workability of PC. They must form a stable and homogeneous mixture with the polymer binder, be low in volatility, and maintain plasticizing effects at normal and low temperatures. Common plasticizers include catapine, alkamon OS-2, melamine-formaldehyde resin, and plasticizer S-3. Esters of phthalic acid, such as dibutyl phthalate (DBP) and dioctylphthalate (DOP), as well as the esters of phosphoric acid (tricresylphosphate), are also commonly used [31].

#### 2.3.2. The Silane Coupling Agent/Silane Treatment

Polymer concrete, being a combination of organic polymer matrix and inorganic aggregates, often exhibits weak adhesiveness. Silane treatment or the addition of silane coupling agents can enhance this adhesiveness. It improves interfacial bonding performance, water and chemical resistance, mechanical properties, and aging resistance, thereby significantly improving the durability and lifespan of the material. Research shows that an increase in silane coupling agents improves chemical resistance while reducing water absorption in PCs containing recycled glass aggregate (RGA) [38]. The most commonly used silane coupling agent is 3-methacryl oxypropyl trimethoxy silane [69,72]. Depending on their suitability for specific resins as recommended by manufacturers, two main silane coupling agents are utilized: Dynasil DEMO (γ-methacryloxy propyl trimethoxy silane) for polyester resin and Dynasil AMEO (γ-amino propyl triethoxy silane) for epoxy resin [5]. Ymethacryl oxypropyl trimethoxy silane has been used for modifying PC [7]. However, the study shows that only enough silane is required to wet all aggregate particles. Less than this amount results in insufficient wetting, while more than this amount has no further benefits. Better strength improvements are obtained by pre-treating either the resin or the aggregates with the silane rather than by adding the silane directly to the polymer concrete mix [77].

## 3. Review of Polymer Concrete and Application of Polymer Concrete

### 3.1. Review of Polymer Concrete Research

Since polymer concrete has been implemented, there has been much research on it, and there are also many review papers that review the development of PCs, showing the repair methods, summarizing the techniques, discussing and comparing the properties of PCs with different resins, and so on. Some review papers on polymer concrete are shown in Table 3.

According to this research, we find that polymer concrete (PC) has been extensively reviewed over the years, with each study contributing to a deeper understanding of its properties, applications, and development. Collectively, these reviews illustrate the significant progress made in understanding and optimizing polymer concrete, highlighting its evolution from a novel material to a widely recognized and utilized composite in the construction industry. Notably, no universal mathematical model currently exists for predicting polymer concrete’s damping and mechanical properties that simultaneously achieves high efficiency, accuracy, minimal input parameters, and low computational cost. Most existing models require extensive experimental datasets. Among available options, XGBoost and DNNs demonstrate the strongest predictive performance (highest R^2^ values) with relatively low testing costs, making them the most promising candidates [88].

In addition to these review papers, books like [2,42] further enrich the understanding of polymer concrete. The book in [2] reports the status of advanced polymer and silicate polymer concretes and compounds. It examines their physical, mechanical, and technological properties; their behavior upon exposure to harsh environmental factors; and the issues of durability and reliability. Furthermore, the book [65] provides a comprehensive study of polymer concrete (PC), covering its historical perspectives, classification, applications, advantages, disadvantages, material effects, fabrication methods, property testing standards, and future applications. Together, these sources contribute significantly to the comprehensive knowledge base of polymer concrete, fostering its continuous development and broader adoption in various engineering and construction applications.

### 3.2. Applications of Polymer Concrete

**Figure 8:**
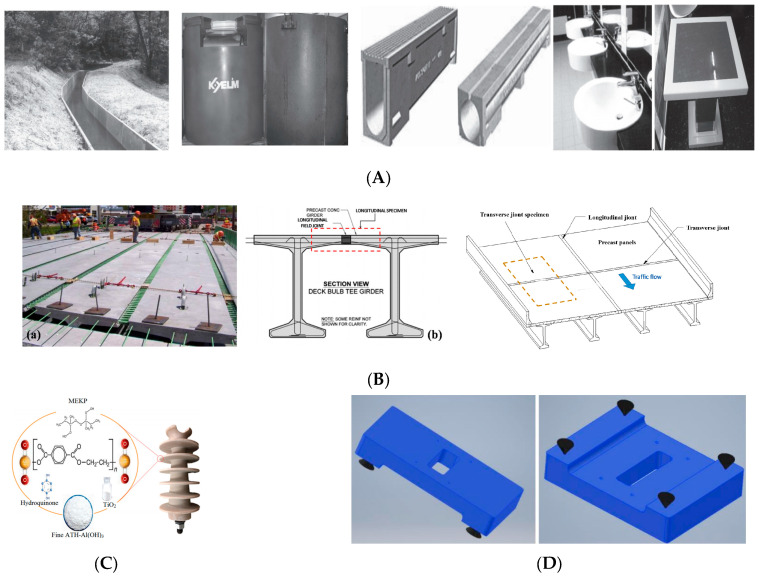

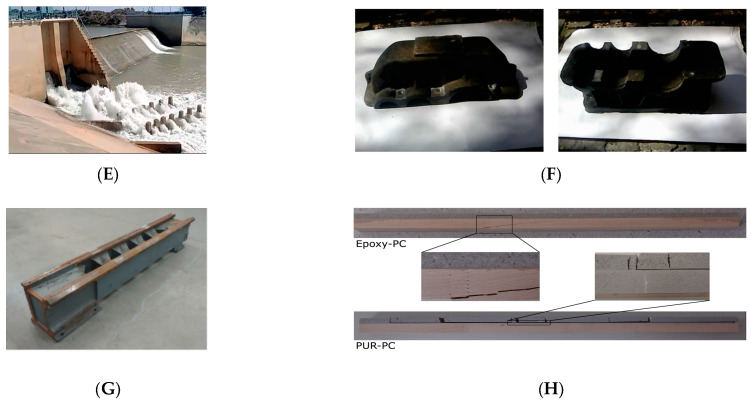
Some photos of the applications of PC. (**A**) Flume, container, pre-slope trench, and artificial marble products [80]. (**B**) Poly methyl methacrylate polymer concrete for bridge deck bulb tee girder longitudinal field joints, (a) construction of a DBT girders bridge of Route 31 Bridge in Lyons (b) schematic of field joints and test specimen [90,91]. (**C**) PC line-post insulators [92]. (**D**) Machine tool bed [93]. (**E**) PC use in hydraulics [94]. (**F**) Manufacture of gear cases and pumps [95]. (**G**) Body of lath [96]. (**H**) Timber concrete composites [97].

Today, the use of PCs is increasing rapidly in many industries. According to recent research by Global Market Insights, the polymer concrete market is expected to surpass a market value of over USD 750 million by the year 2025 while registering a CAGR of 7.9% during the forecast 2022–2030 [89]. Polymer concrete was developed as a replacement for cement concrete in some specific applications and was used as early as 1958 in the US to produce building cladding. It was later implemented as an alternative to other substances, such as metal alloys; for example, cast iron for machine beds. Due to fast curing time being one of the main advantages of PCs, polymer concrete was widely used for repair, strengthening, and protection, such as maintenance applications like highway pavement, bridge overlay, and floor covering. Precast polymer concrete has been used to produce a variety of products like acid tanks, manholes, drains, highway median barriers, and so forth. According to the book [65], the applications can be classified as electrical and communication, overlay and coatings, repair material, water supply and drainage, agricultural irrigation facilities, container, architectural use, building and construction, machine parts, high-pressure and -temperature media, hydraulic structures, transportation, and 3D printers. Some photos of the applications of PC are shown in Figure 8.

PC can be implemented in complex concrete sleepers of high-speed train systems because it reduces the radiation of the rolling noise [98], but this application was not well justified since the rolling noise frequency spectra often appear at a relatively low-frequency range; most noise issues related to high-speed rail systems tend to be associated with high-frequency noises [98]. In addition to the applications previously mentioned, recent years have seen novel uses, such as bonding different materials, such as steel rods, in Douglas fir roundwood specimens with PC [99]. Nine categories of potential materials for lunar infrastructure were identified, with polymer concrete presenting the most feasible approach for the initial infrastructure [100]. PC has been used in the construction of electrical insulators since the 1970s, and now there have been some notable results, such as a power frequency withstand voltage of 75 kV in wet conditions, a dry lightning impulse voltage of 161 kV, and a puncture withstand voltage of 195 kV. All these could provide significant guidance for insulation design and anti-pollution works related to line-post insulators used in electrical distribution networks, and open up new applications for PC insulators used in the electrical industry [92]. Furthermore, its use as a 3D printing material is becoming increasingly noticed. The paper [29] focuses on the evaluation of polymer concrete as a three-dimensional (3D) printing material, and the results suggest that PC is a good fit for 3D printing, with little to no degradation caused by the process. Layer adhesion was shown to be excellent, with a negligible effect on the finished part for the longitudinal orientation.

These applications highlight the versatility and growing importance of polymer concrete across various sectors, from construction and infrastructure to advanced manufacturing and space exploration. As research and technological advancements continue, the scope for PC applications is expected to expand further, driven by PC’s unique combination of properties and performance benefits.

### 3.3. Sustainability of Polymer Concrete

#### 3.3.1. Reducing Landfill Dependency

Polymer concrete (PC) demonstrates significant environmental advantages by effectively incorporating various waste materials. Industrial byproducts such as fly ash, slag, silica fume, and ceramic slurry, along with agricultural residues like coconut husk, rice husk, and hemp fibers, serve as sustainable fillers and reinforcements in PC formulations. The use of phosphogypsum is also applied as an environmentally friendly filler in polymer concrete [101,102]. Waste-derived materials not only divert substantial volumes of industrial and agricultural waste from landfills but also reduce reliance on virgin raw materials, lowering the overall environmental impact of construction [52]. The compatibility and the optimal dosages between the components were studied, the amount of resin used was minimized, and the mineral and organic waste was maximized, contributing to the valorization of agricultural and quarry waste, reducing the impact on the environment, and improving production costs [103]. While different waste materials contribute distinct properties—with industrial wastes generally enhancing durability and natural fibers improving toughness—their incorporation consistently supports sustainability goals, even when mechanical performance trade-offs exist. For instance, textile fibers may not increase compressive strength, but they effectively mitigate brittleness [104], while machining chips can partially replace conventional fillers despite potential minor reductions in strength parameters [17].

#### 3.3.2. Reducing Carbon Emission Mitigation

Another critical sustainability benefit of polymer concrete lies in its ability to encapsulate waste materials within its microstructure, preventing their release into the environment while avoiding the high carbon footprint associated with Portland cement production. Cement manufacturing is responsible for approximately 8% of global CO_2_ emissions. In contrast, polymer concrete relies on resin binders (e.g., epoxy, polyester, or vinyl ester); there is usually no reactivity between the surrounding polymer matrix and aggregate particles, resulting in a significantly lower carbon footprint [19].

When waste materials such as fly ash, slag, or ceramic residues are embedded in the polymer matrix, they can be safely locked within the polymer structure, preventing the leaching of harmful substances into the environment. Moreover, some waste materials, such as biochar, can contribute to carbon-negative concrete by storing carbon that would otherwise be released through decomposition or incineration. This makes polymer concrete an attractive option for sustainable construction in line with global decarbonization goals [19,20].

As described above, the sustainability of polymer concrete is significantly enhanced through the strategic use of waste materials as fillers, which reduces landfill dependency and promotes a circular economy. Additionally, by eliminating cement and encapsulating waste within its microstructure, PC offers a viable pathway for carbon emission reduction in the construction sector. Future research should focus on optimizing waste-based filler compositions to further improve mechanical performance while maximizing environmental benefits.

## 4. Conclusions

In conclusion, polymer concrete (PC) has established an irreplaceable position in the field of engineering construction since its inception due to its excellent mechanical strength, rapid curing characteristics, outstanding chemical corrosion resistance, and environmental durability. This article systematically studies the components of PC, including resin matrix, fillers, fibers, and nanomaterials, as well as various additives, and reveals the mechanism of each component in customizing material properties. Resins, such as polyester and epoxy, form the matrix that binds the aggregate and fillers together, providing the necessary mechanical strength and chemical resistance. Fillers and fibers further enhance the structural integrity and durability of the material, while nanofillers contribute to improved mechanical properties and performance. Advances in materials science have enabled the formulation of PC variants that excel in diverse conditions, from harsh industrial settings to delicate architectural applications.

Throughout the development and application of PC, it can be seen that it has been widely used in global infrastructure projects. From highways and bridges to electrical insulators, 3D-printed structures, and potential materials for lunar infrastructure, PC’s versatility and durability have positioned it as a preferred choice for engineers and architects alike. Market analysis shows that the global PC market will maintain a strong growth trend. Ongoing research and development are expanding PC’s capabilities, ensuring its role as a key material in sustainable construction and resilient infrastructure. By leveraging its inherent strengths, such as durability, efficiency, and environmental benefits, PC is set to be applied in the future of construction, providing high-performance, eco-friendly solutions.

Looking towards the future, polymer concrete (PC) technology will focus on several key innovative directions to drive sustainable development in construction. Green manufacturing technology will significantly reduce environmental footprints through recyclable materials and low-carbon formulations across the material lifecycle. Advanced manufacturing technologies like 3D printing will enable the rapid customized production of complex components, complemented by hybrid manufacturing approaches that optimize economic benefits for large-scale projects. Specialized formulations will be developed to enhance material durability under extreme climate conditions, ensuring reliable performance in challenging environments. Importantly, the establishment of comprehensive material recycling and regeneration systems will promote circular economy development within the construction industry. These interconnected advancements will collectively position PC as a material for sustainable infrastructure development.

## Figures and Tables

**Table 1:** Commonly used polymeric resins, along with their disadvantages.

Commonly Used Polymeric Resins	Most Significant Disadvantages and Their Viscosity Values
epoxy resin [30]	Expensive, high viscosity (11–14 Pa·s at 25 °C)
polyester resin [31]	Toxicity in the uncured state (0.4 Pa·s at 25 °C)
vinyl ester resin [32]	High shrinkage, harder to handle, deterioration due to thermally induced cracks and bond failure between concrete and overlay; (0.2–0.35 Pa·s at 25 °C)
furan resins [33]	Short-term pot life, toxicity in the uncured state, high self-heating temperature causing significant thermal stresses, affecting strength (0.02~0.05 Pa·s at 20 °C)
polyurethane resins [34]	The thermal resistance is generally poor; prolonged exposure to elevated temperatures may lead to softening, strength degradation, or decomposition (0.2–2 Pa·s at 25 °C)
Poly(methyl methacrylate) [8]	Low flash point of the MMA monomer, posing safety problems (0.56 Pa·s at 20 °C)
Carbamide (urea-formaldehyde) [35]	Relatively low physical and mechanical properties (lower than 0.1 Pa·s at 25 °C)

**Table 2:** Various agents used in polymer concrete.

Type of Resin/PC	Agent
epoxy resin [30].	harder: aliphatic amine
epoxy resin ROPOXID 701 [9]	curing agent (Harder:): ROMANID 407
polyester resin [66]	curing agents: Akcobalt 6% (cobalt 2-ethylhexanoate mixture) and Akperox A1 (Methyl Ethyl Ketone Peroxide-MEKP)
polyester resin [31]	plasticizer: dibutyl-phthalate, chlorinated paraffin, technical glycerin, and engine oil
polyester resins [67]	superplasticizer: CONPLAST SP430
polyester resin (MEKP-NR20) [68]	initiator: Methyl ethyl ketone peroxide (MEKP) in dimethyl phthalate (DMP)
unpromoted polyester-MMT PC [69]	initiators: 0.3% weight of Benzoyl peroxide
unsaturated polyester resin [70].	initiator promoter: methyl ethyl ketone peroxide (MEKP) and cobalt naphthenate (CoNp)/Benzoyl peroxide (BPO) and *N*,*N*-diethyl aniline (NNDA)
unsaturated polyester resin [71]	hardener: 2 wt% methyl ethyl ketone peroxide (MEKP) accelerator: 1 wt% cobalt octoate
acrylic polymer concrete [72]	initiator: Benzoyl peroxide (BPO) catalyst: N, N-Dimethylaniline and *N*,*N*-Dimethyl-p-toluidine auxiliary accelerator: MAA, a type of polar monomer cross-linking agent: TMPTMA coupling agent: silane (3-methacryl oxypropyl trimethoxy silane)
acrylic resin [73]	initiator: benzoyl peroxide (BPO) accelerator: *N*,*N*-dimethylaniline (DMA) auxiliary accelerator: methacrylic acid (MAA)
acrylic resin [10]	initiator: benzoyl peroxide(BPO) promoter: *N*,*N*-dimethyl-p-toluidine(DMT) cross-linking agent: trimethylolpropane trimethacrylate(TMPTMA), a highly reactive tri-functional monomer, promoting hardening reactions by free radical polymerization
vinyl ester [32]	initiator: benzoyl peroxide(BPO) (function as harder) with dimethylaniline and cobalt naphthenate(function as an accelerant)
glycerol methacrylate/styrene polymer concrete [74]	initiators: methyl acetoacetate peroxide (MAAPO) accelerators: cobalt naphthenate (CoN)
furfuryl alcohol(FA) [75]	initiators: benzenesulfonic acid, p-toluenesulfonic acid, trichloroacetic acid, a, a, a-trichlorotoluene, a, a-dichlorotoluene, and a-chlorotoluene

**Table 3:** Review papers on polymer concrete.

Year/Author	Main Review Work	Brief Findings
1985 [78] Gunasekaran, M	Development of PC for high-voltage insulation applications, highlighting the progress made in the previous decade and emphasizing its versatility as a composite material.	PC insulators perform well in the field and are highly competitive with cycloaliphatic epoxy and standard electrical porcelain.
1994 [79] Gunasekaran, M	Performance of different types of polymer concrete insulation systems, discussing their durability and opportunities for improvement in field applications.	PC insulation can replace porcelain and is versatile for both insulating and structural applications in the electric power industry.
2010 [80] Yeon, K.-S.	Physical and mechanical properties, product applications, economic analysis, and environmental impacts of PC. The prospects of polymer concrete in the construction market.	Besides mechanical strengths, it turned out that PC has beneficial performance as construction materials in overall properties.
2011 [81] Allahvirdizadeh, R	Discusses concrete repair methods and materials, analyzes different effects on PC behavior, evaluates wear resistance, and provides repair scenario examples.	PC is not suitable for high temperatures but is resistant to freeze–thaw cycles and deicing solutions, requires careful selection, and is allowed for use in inaccessible cases.
2013 [1] Bedi, R.	Summarizes the efforts on selecting ingredients, optimizing processing parameters, controlling curing conditions, and their impacts on the mechanical properties of PC.	Epoxy PC outperforms polyester in key properties. Using locally available materials reduces costs, while optimizing aggregate mix maximizes strength. Aggregate moisture below 0.5% and adding silane coupling agents are both benefits.
2014 [82] Bedi, R.	Ingredient selection, processing parameters, curing conditions, and their influence on the mechanical properties of the material.	Resin dosage ranges from 10% to 20% by weight in PC. Assisted drying and the use of gap-graded aggregates are beneficial, while 1% silane relative to resin weight yields optimal outcomes.
2015 [83] Momtazi, A.S.	Modern construction techniques and recent research advancements in PC applications and improvements.	Epoxy polymer concrete can store anti-ice fluids and then release them during snowfall and severe weather conditions (frost).
2016 [84] Kumar, R.	Formulations and properties of epoxy and polyester-based polymer concrete, highlighting mechanical, thermal, and water resistance properties.	The shift to polyfurfuryl alcohol (PFA) as a sustainable bioresin for PC offers superior chemical resistance compared to traditional thermoset polymers.
2018 [85] Ali-Askari, K.O	Compares features between PC and OPC, examining compound system functions and presenting various applications of polymer concrete.	PC displays shapeability after loading, has a lower elasticity modulus, exhibits nearly double the linear thermal expansion coefficient of ordinary concrete, and has lower penetrance and higher chemical resistance.
2018 [86] Venkatesh, B.	Discusses resin and fiber proportions, mechanical properties, mix design, advantages, and applications of resin-based PC.	The addition of fly ash as filler material in polymer resin concrete results in an economical mixture, and the ductility can be improved with increasing resin content.
2022 [4] Nodehi, M.	Fresh, mechanical, and durability properties of PC; comparative analyses of different resins and their performance results.	PC provides superior strength, corrosion resistance, and chemical durability. Adjusting fillers and cementitious materials allows customization of its flowability and properties.
2023 [87] Hassani Niaki	Evaluate fracture properties of PC, exploring governing equations, fracture patterns, and parameters affecting fracture behaviors.	Exposure to high temperatures, thermal cycles, and chemical solutions, as well as exposure to atmospheric conditions, can have destructive effects on the fracture properties of PC.
2024 [88] Aleksandr, P	Polymer Concretes Based on Various Resins: Modern Research and Modeling of Mechanical Properties	The most promising polymers for use in the field of road surface repair are polymer concretes with poly(meth)acrylic resins. The most adequate and productive models are the deep machine learning model and the extreme gradient boosting model.

## Data Availability

No new data were created or analyzed in this study. Data sharing is not applicable to this article.
